# Development and internal validation of a clinical prediction model for postoperative venous thromboembolism after neurosurgical intervention in patients with traumatic brain injury

**DOI:** 10.3389/fneur.2026.1953953

**Published:** 2026-10-12

**Authors:** Dongping Li, Jianhui Chen, Chunfa Weng, Jianhuang Huang

**Affiliations:** 1 Department of Critical Care Medicine, Affiliated Hospital of Putian University, Putian, Fujian, China; 2 Department of Neurosurgery, Affiliated Hospital of Putian University, Putian, Fujian, China

**Keywords:** clinical prediction model, internal validation, interpretability, logistic regression, postoperative complications, traumatic brain injury, venous thromboembolism

## Abstract

**Background:**

Venous thromboembolism (VTE) is a critical complication after traumatic brain injury (TBI) surgery. Conventional risk assessment tools were not developed for neurosurgical trauma populations, limiting their applicability.

**Objective:**

To develop and internally validate an interpretable clinical prediction model for postoperative VTE in TBI surgery patients.

**Methods:**

This retrospective cohort study enrolled 1,450 TBI patients who underwent surgery (2016–2024). Seven predictors were selected using Recursive Feature Elimination. Six machine learning algorithms were compared, with logistic regression (LR) selected as the final model. Performance was evaluated using AUC-ROC, calibration, and decision curve analysis.

**Results:**

Among 1,450 patients, 205 (14.1%) developed postoperative VTE. The LR model achieved AUC-ROC of 0.868 (95% CI: 0.815–0.920) with good calibration (Brier score: 0.085). Seven predictors were identified: age, Barthel index, ASA classification, multiple surgeries, general anesthesia, limb AIS score, and serum magnesium. Compared with Caprini score (AUC: 0.686), the model showed improved discrimination (ΔAUC = 0.182; NRI: 0.559).

**Conclusion:**

An interpretable model based on seven routinely available variables demonstrated promising performance for postoperative VTE risk stratification in TBI surgery patients. A nomogram and web-based calculator are provided for clinical use.

## Introduction

Venous thromboembolism (VTE), encompassing deep vein thrombosis (DVT) and pulmonary embolism (PE), remains a leading cause of preventable mortality among hospitalized patients (1, 2). Globally, an estimated 10 million individuals experience VTE annually, with PE ranking among the top causes of cardiovascular death in the United States (3). Patients with VTE face prolonged hospitalization, increased 30-day readmission rates, and delayed recovery (4).

Traumatic brain injury (TBI) affects approximately 50 million individuals globally each year and is an independent risk factor for VTE through multifactorial pathophysiological mechanisms (5, 6). Patients with TBI who undergo surgical intervention face particularly elevated VTE risk due to venous stasis from general anesthesia and intraoperative immobilization, postoperative mobility restrictions, tissue injury-induced inflammation and coagulation pathway activation, and neurological impairment that limits early rehabilitation (7). Studies indicate that patients with multitrauma and TBI exhibit significantly higher VTE incidence compared to those without TBI, attributable to disease complexity and delayed early interventions (2, 8). However, thromboprophylaxis decisions in patients with TBI remain uniquely challenging: clinicians must carefully balance the risk of thromboembolism against potential intracranial hemorrhagic complications, often leading to delayed or omitted anticoagulant prophylaxis in high-risk surgical patients (9).

The special characteristics of TBI patients limit the applicability of conventional VTE prediction tools. First, TBI patients have distinct pathophysiology compared to patients with cancer, medical illnesses, or general surgical conditions--the two populations in which most existing VTE models were developed. Second, neurosurgical patients face an elevated bleeding diathesis in the context of brain parenchyma and subdural/subarachnoid hematomas, making risk assessment more nuanced than simple thrombosis scoring. Third, established VTE prediction instruments such as the Caprini and Padua scores rely heavily on subjective historical risk factors and fail to account for anatomical factors specific to neurosurgical trauma populations, including limb injury severity, preoperative functional dependence, and perioperative biochemical parameters (10, 11). Therefore, a precise risk stratification model tailored to this population may assist clinicians in identifying patients who require intensified surveillance, early mobilization strategies, or individualized VTE prevention strategies while minimizing unnecessary hemorrhagic complications (12).

Machine learning (ML) provides a flexible framework for integrating heterogeneous clinical variables while maintaining interpretability when combined with transparent feature selection methods (13). In recent years, ML-based prediction models have demonstrated utility in perioperative risk stratification across diverse surgical contexts (14); however, their application to postoperative VTE prediction in neurosurgical trauma remains underexplored. Moreover, in moderately sized datasets with limited event numbers, overly complex models are prone to overfitting, potentially undermining generalizability despite excellent cross-validation performance (15). Selecting an appropriately balanced model that prioritizes both discrimination and interpretability is therefore essential for clinical translation. Notably, there is currently a lack of a postoperative, neurosurgical trauma-specific model that incorporates both functional impairment and injury-related anatomical burden--dimensions particularly relevant to TBI patients that are not captured by general surgical risk scores.

An important consideration in prediction model development is the balance between data-driven variable selection and clinical pathophysiological reasoning. Purely algorithmic feature selection without clinical grounding may identify statistically associated but biologically implausible predictors, reducing clinician trust and generalizability. Conversely, relying solely on *a priori* clinical knowledge may miss novel associations. We therefore adopted a hybrid approach: candidate predictors were predefined based on established VTE pathophysiology (encompassing immobility, injury severity, surgical burden, coagulation, and systemic inflammation domains) and clinical availability, followed by RFE as a secondary dimensionality reduction strategy to optimize model parsimony within this clinically grounded candidate set. Accordingly, the objective of this study was to develop and internally validate an interpretable, multidimensional prediction model for postoperative VTE in surgical TBI patients. We hypothesized that integrating conventional host vulnerability (age, ASA status), functional dependence (preoperative Barthel index), anatomical injury burden (Limb AIS score), perioperative trauma (anesthesia type, surgical frequency), and biochemical markers of endothelial/hemostatic stability (serum magnesium) would provide superior, individualized risk stratification over generic risk scores.

## Methods

### Study design and reporting

This retrospective cohort study was conducted at Affiliated Hospital of Putian University and reported in accordance with the TRIPOD statement and TRIPOD+AI extension (16, 17). The TRIPOD+AI checklist is available as Supplementary material.

### Study population and surgical procedures

This study consecutively enrolled patients with traumatic brain injury (TBI) who underwent surgical intervention at Affiliated Hospital of Putian University between October 2016 and December 2024. TBI was defined as any brain injury resulting from external mechanical force, confirmed by CT imaging. Surgical intervention included craniotomy for hematoma evacuation, decompressive craniectomy, skull fracture repair, and intracranial pressure monitor placement.

Surgical interventions were classified into four mutually exclusive categories based on the primary operative intent: craniotomy for hematoma evacuation (epidural, subdural, or intraparenchymal hematoma), decompressive craniectomy (with or without hematoma evacuation), debridement and elevation of depressed or open skull fractures, and isolated invasive intracranial pressure (ICP) monitor placement.

Of note, while invasive ICP monitor placement is frequently performed at the bedside in some international health systems, institutional surgical governance and national neurotrauma infection control guidelines in China mandate that all invasive ICP monitor insertions (including intraparenchymal probe placement and external ventricular drain [EVD] insertion via burr hole) be conducted within the sterile, laminar-flow operating theater under formal surgical scheduling.

Regarding anesthesia modalities, 87.9% of patients (*n* = 1,274) received general endotracheal anesthesia, while 12.1% (*n* = 176) underwent surgery under local scalp infiltration anesthesia with or without monitored anesthesia care (MAC). All major craniotomies (*n* = 782) and decompressive craniectomies (*n* = 384) were strictly conducted under general anesthesia. Non-general anesthesia was utilized exclusively in the operating room for selected less-invasive procedures: 102 isolated ICP monitor placements in cooperative or lightly comatose patients with stable spontaneous airways, and 74 limited, non-complex depressed skull fracture debridements.

Inclusion criteria: (1) confirmed TBI diagnosed by CT; (2) surgical management during hospitalization; (3) age ≥18 years. Exclusion criteria: (1) age <18 years; (2) no surgical intervention; (3) missing >20% of essential variables; (4) hospital stay <72 h; (5) pre-surgical VTE.

### Outcome definition

The primary outcome was postoperative VTE (DVT or PE) diagnosed during hospitalization after surgical intervention. The prediction time point was defined as 24 h after surgery completion. VTE diagnosis was established using compression Doppler ultrasonography for DVT and CT pulmonary angiography for PE when clinically suspected. Routine asymptomatic screening was not performed; therefore, the outcome represents clinically apparent VTE.

### Perioperative VTE prophylaxis protocol

Standard institutional practice mandated mechanical thromboprophylaxis using intermittent pneumatic compression (IPC) sleeves upon admission unless contraindicated by severe lower extremity fractures. Pharmacologic thromboprophylaxis predominantly utilized low-molecular-weight heparin (LMWH; enoxaparin 40 mg once daily subcutaneously) or unfractionated heparin (UFH; 5,000 IU every 8–12 h in patients with creatinine clearance < 30 mL/min). Chemoprophylaxis initiation was contingent upon demonstration of intracranial hemorrhagic stability on repeat cranial CT (typically obtained 24–72 h postoperatively). In real-world clinical practice, the timing of initiation varied according to attending neurosurgeon discretion and perceived hemorrhagic risk; overall, 41.2% of patients received chemoprophylaxis during hospitalization, initiated at a median of 4.2 days (IQR, 3.0–6.5 days) post-injury. No chemoprophylaxis was administered within the first 24 postoperative hours.

### Data collection

Clinical data were collected from the electronic medical record system by trained research assistants using standardized forms. Predictor variables encompassed six domains: (1) demographics; (2) preoperative comorbidities; (3) injury characteristics including Glasgow Coma Scale, ISS, and AIS scores; (4) biochemical parameters including serum magnesium; (5) preoperative interventions; and (6) surgical and anesthesia details. The Barthel index was assessed preoperatively to evaluate functional status. Detailed variable definitions are provided in Supplementary Table S1.

### Feature selection

Candidate predictors were predefined based on VTE pathophysiology and clinical availability, organized into domains corresponding to Virchow’s triad. Recursive Feature Elimination (RFE) with logistic regression was used as a secondary dimensionality reduction strategy within cross-validation folds. Seven features were selected: age, Barthel index, ASA classification ≥3, multiple surgeries, general anesthesia, limb AIS score, and serum magnesium levels.

Injury severity was comprehensively assessed at baseline using the GCS, the Injury ISS, and AIS scores across all anatomical domains: Head, Face, Thorax, Abdomen, Spine, and Extremities/Pelvic girdle. During the RFE procedure, non-extremity AIS scores (Thorax, Abdomen, Spine) and composite ISS were eliminated due to lower relative feature weights and collinearity. Head AIS exhibited minimal discriminative variance because severe cranial injury was an entry criterion for neurosurgical intervention. Limb AIS was retained as the optimal anatomical injury metric, capturing both severe localized endothelial injury and immobilization-induced venous stasis.

### Model development

Six machine learning algorithms were evaluated: logistic regression (LR), random forest (RF), gradient boosting decision tree (GBDT), extreme gradient boosting (XGBoost), support vector machine (SVM), and categorical boosting (CatBoost). Missing data were handled using multiple imputation by chained equations (MICE). All preprocessing steps were performed within cross-validation pipelines to prevent information leakage. Detailed algorithm configurations and hyperparameter optimization procedures are provided in Supplementary Table S2.

The final model was selected based on discrimination (AUC-ROC), calibration (Brier score, calibration slope), clinical utility (DCA), interpretability, and stability. Logistic regression was selected due to its comparable discrimination (AUC-ROC: 0.868), favorable calibration (slope: 0.95; Brier score: 0.085), superior interpretability, and stable performance between cross-validation and validation sets.

A nomogram was constructed based on the final model, with points allocated proportionally to regression coefficients. A web-based calculator is publicly available at https://heroic-youtiao-ecdd4b.netlify.app/. The prediction equation is provided in the Results section.

### Model evaluation

Model performance was evaluated using AUC-ROC with 95% confidence intervals (DeLong method), calibration metrics (Brier score, calibration slope, calibration-in-the-large), and decision curve analysis. Bootstrap resampling with 1,000 iterations was performed to assess internal validity and compute optimism-corrected performance metrics.

### Comparison with established risk scores

While the Padua Prediction Score and the Caprini Risk Assessment Model are both widely recognized VTE tools, they are tailored to fundamentally different populations: Padua was developed exclusively for hospitalized internal medicine (non-surgical) patients and omits surgical trauma variables, whereas Caprini was specifically designed for surgical and trauma cohorts. Because our cohort comprises surgical neurotrauma patients, the Caprini score was selected as the sole appropriate clinical comparator. The Caprini score was calculated for all patients in the validation set using all available clinical variables. Incremental predictive value was quantified using category-based net reclassification improvement (NRI) and integrated discrimination improvement (IDI), with risk categories defined as: low (<10%), intermediate (10–30%), and high (>30%).

### Magnesium analysis

The association between serum Mg2 + and VTE was explored using dose–response analysis (quartile stratification), restricted cubic spline regression (4 knots at 5th, 35th, 65th, and 95th percentiles), and adjusted logistic regression. This analysis was considered exploratory and hypothesis-generating.

### Sensitivity analyses

Sensitivity analyses were performed to assess: (1) impact of different imputation methods; (2) different train-validation split ratios; (3) SMOTE oversampling for class imbalance; and (4) regularization strength. Detailed results are provided in Supplementary materials.

### Statistical analysis

All analyses were performed using Python 3.10.12 with scikit-learn, statsmodels, XGBoost, CatBoost, imbalanced-learn, and SciPy. Continuous variables were presented as medians (IQRs) and compared using Mann–Whitney U test. Categorical variables were presented as counts (percentages) and compared using chi-square or Fisher exact test. *p* < 0.05 was considered statistically significant. More methodological details are provided in the Supplementary materials.

### Model availability

The prediction equation, nomogram, and web-based calculator are provided as Supplementary materials. The web-based calculator is publicly accessible at: https://heroic-youtiao-ecdd4b.netlify.app/. The source code is available from the corresponding author on reasonable request.

## Results

### Study population

From October 2016 to December 2024, a total of 1,450 patients with TBI who underwent surgical intervention were enrolled in this study (Figure 1). Among these patients, 205 (14.1%) developed postoperative VTE during hospitalization.

**Figure 1:**
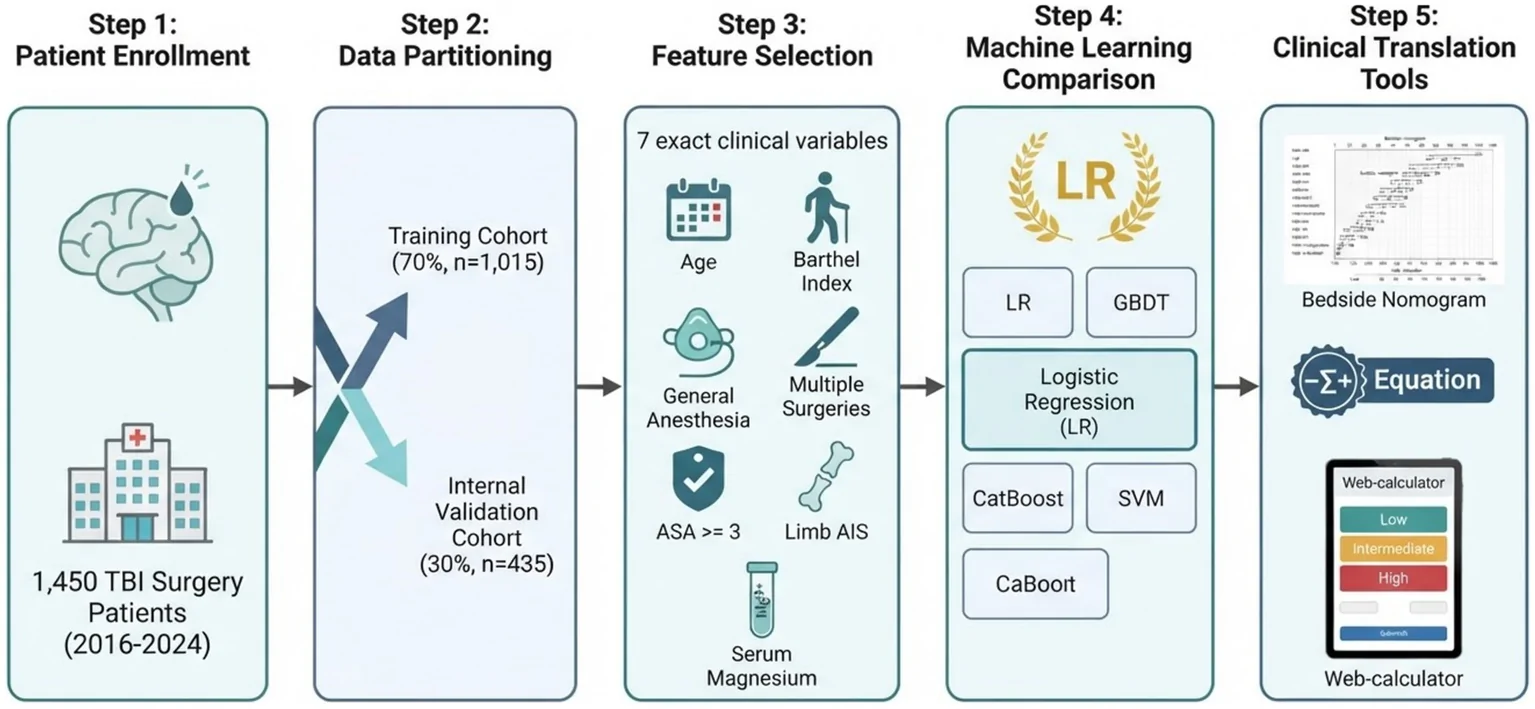
Study flow and model development pipeline. The diagram illustrates patient enrollment (1,450 TBI surgery patients), data partitioning (70% training, 30% validation), candidate variable definition based on VTE pathophysiology domains, RFE feature selection within cross-validation folds, six-algorithm comparison, final model selection (LR), and output tools (nomogram, prediction equation, web calculator). AIS, Abbreviated Injury Scale; ASA, American Society of Anesthesiologists; BI, Barthel index; DCA, decision curve analysis; LR, logistic regression; Mg2+, serum magnesium; RFE, recursive feature elimination; ROC, receiver operating characteristic; SMOTE, synthetic minority oversampling technique; TBI, traumatic brain injury; VTE, venous thromboembolism.

### Baseline characteristics

Table 1 presents the baseline characteristics of the study population stratified by VTE status. Patients with VTE were significantly older (median age: 54 vs. 50 years; *p* = 0.002), had higher ASA classification > = 3 rates (80.5% vs. 59.9%; *p* < 0.001), higher rates of multiple surgeries (38.0% vs. 25.0%; *p* < 0.001), higher limb AIS scores (median: 3 vs. 0; *p* < 0.001), and lower serum Mg2 + levels (median: 0.81 vs. 0.86 mmol/L; *p* < 0.001). Missing data for key variables are summarized in Supplementary Table S3 (Supplementary Figure S1).

**Table 1:** Baseline characteristics of patients stratified by VTE status.

Variable	Total (*N* = 1,450)	No VTE (*n* = 1,245)	VTE (*n* = 205)	*p*-value
Age (y), median (IQR)	51 (43–59)	50 (42–59)	54 (47–59)	0.002
Weight (kg), median (IQR)	61 (55–68)	61 (55–68)	61 (55–67)	0.784
Barthel index, median (IQR)	10 (0–40)	10 (0–40)	10 (0–20)	<0.001
Male, *n* (%)	1,052 (72.6)	897 (72.0)	155 (75.6)	0.330
ASA classification > = 3, *n* (%)	911 (62.8)	746 (59.9)	165 (80.5)	<0.001
Multiple surgeries, *n* (%)	389 (26.8)	311 (25.0)	78 (38.0)	<0.001
General anesthesia, *n* (%)	1,274 (87.9)	1,086 (87.2)	188 (91.7)	0.088
Limb AIS score, median (IQR)	0 (0–3)	0 (0–2)	3 (2–4)	<0.001
Serum Mg2 + (mmol/L), median (IQR)	0.8 (0.8–0.9)	0.9 (0.8–0.9)	0.8 (0.7–0.9)	<0.001
GCS score, median (IQR)	13 (10–15)	13 (10–15)	13 (10–15)	0.553
ISS, median (IQR)	16 (12–19)	15 (12–19)	16 (13–19)	0.526
Hypertension, *n* (%)	202 (13.9)	170 (13.7)	32 (15.6)	0.522
Diabetes, *n* (%)	88 (6.1)	74 (5.9)	14 (6.8)	0.738
Smoking, *n* (%)	441 (30.4)	375 (30.1)	66 (32.2)	0.606
Primary Surgical Procedure, *n* (%)				< 0.001
—Craniotomy for hematoma evacuation	782 (53.9)	671 (53.9)	111 (54.1)	
—Decompressive craniectomy	384 (26.5)	312 (25.1)	72 (35.1)	
—Elevation/repair of skull fracture	168 (11.6)	155 (12.4)	13 (6.3)	
—Isolated ICP monitor placement*	116 (8.0)	107 (8.6)	9 (4.4)	
General anesthesia, *n* (%)	1,274 (87.9)	1,086 (87.2)	188 (91.7)	0.088

^*^Isolated ICP monitor placement was conducted in the operating room under strict aseptic surgical protocols.

AIS, Abbreviated Injury Scale; ASA, American Society of Anesthesiologists; GCS, Glasgow Coma Scale; ICP, intracranial pressure; IQR, interquartile range; ISS, Injury Severity Score; Mg2+, serum magnesium; VTE, venous thromboembolism.

### Feature selection

RFE identified 7 key predictive features from the training set (Figure 2A): Age, Barthel index (BI), ASA physical status classification > = 3, Multiple surgeries, General anesthesia, Limb Abbreviated Injury Scale (AIS) score, Serum magnesium (Mg2+) levels. The standardized regression coefficients of the final LR model are visualized in Figure 2B, showing the relative contribution of each selected feature to VTE risk prediction.

**Figure 2:**
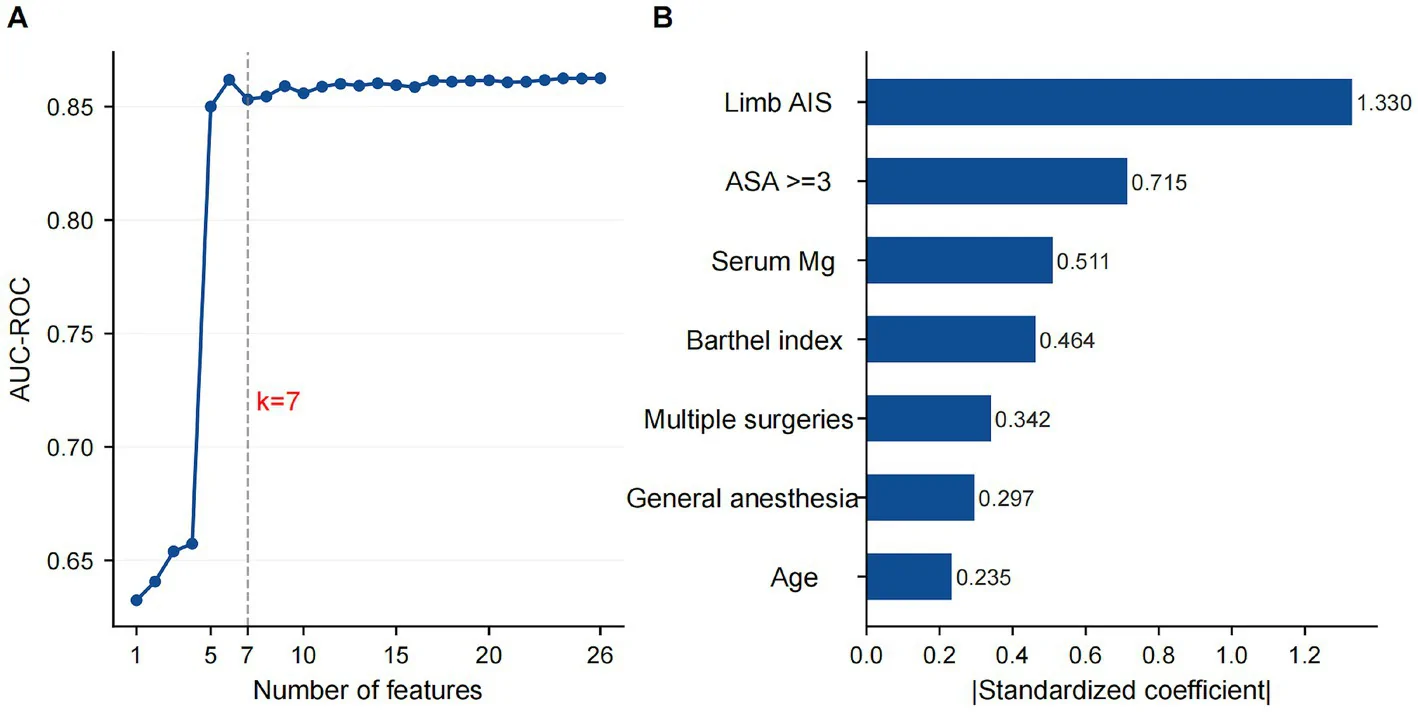
Study characteristics and feature selection. **(A)** Recursive Feature Elimination (RFE) curve showing cross-validation AUC-ROC across different numbers of features, with the optimal 7-feature set highlighted. **(B)** Standardized logistic regression coefficient bar chart showing the direction and magnitude of each selected predictor’s contribution to VTE risk. AIS, Abbreviated Injury Scale; ASA, American Society of Anesthesiologists; AUC-ROC, area under the receiver operating characteristic curve; BI, Barthel index; CI, confidence interval; LR, logistic regression; Mg2+, serum magnesium; OR, odds ratio; RFE, recursive feature elimination; SD, standard deviation; VTE, venous thromboembolism.

### Internal validation set performance

Table 2 presents the performance metrics of all models in the internal validation set (Figure 3A). Although CatBoost achieved the numerically highest AUC-ROC (0.869), LR demonstrated comparable discrimination (AUC-ROC: 0.868) with balanced calibration performance (calibration slope: 0.95; intercept: −0.06; Brier score: 0.085) while maintaining superior interpretability and comparable net clinical benefit on DCA. CatBoost showed slightly higher discrimination, whereas LR demonstrated a more favorable balance between discrimination, calibration, and clinical interpretability--factors collectively more important than AUC alone for clinical prediction model deployment. Notably, ensemble models such as RF and CatBoost, which showed strong cross-validation AUC values (~0.89), exhibited larger performance declines in the validation set, whereas LR showed consistent performance between cross-validation and validation, suggesting improved performance stability in this moderate-sized cohort with limited event numbers.

**Table 2:** Model performance in predicting postoperative VTE in the validation set.

Model	AUC-ROC (95% CI)	Accuracy	Sensitivity	Specificity	PPV	NPV	F1-score	Brier score
LR	0.868 (0.815–0.920)	0.868	0.852	0.783	0.391	0.970	0.536	0.085
CatBoost	0.863 (0.810–0.915)	0.863	0.738	0.877	0.495	0.953	0.593	0.089
GBDT	0.861 (0.808–0.913)	0.861	0.902	0.676	0.313	0.977	0.465	0.086
XGBoost	0.864 (0.812–0.917)	0.864	0.852	0.754	0.361	0.969	0.507	0.083
RF	0.849 (0.797–0.901)	0.849	0.787	0.797	0.387	0.958	0.519	0.158
SVM	0.852 (0.800–0.904)	0.852	0.820	0.797	0.397	0.964	0.535	0.090

AUC-ROC, area under the receiver operating characteristic curve; CatBoost, categorical boosting; CI, confidence interval; F1, F1-score; GBDT, gradient boosting decision tree; LR, logistic regression; NPV, negative predictive value; PPV, positive predictive value; RF, random forest; SVM, support vector machine; VTE, venous thromboembolism; XGBoost, extreme gradient boosting.

**Figure 3:**
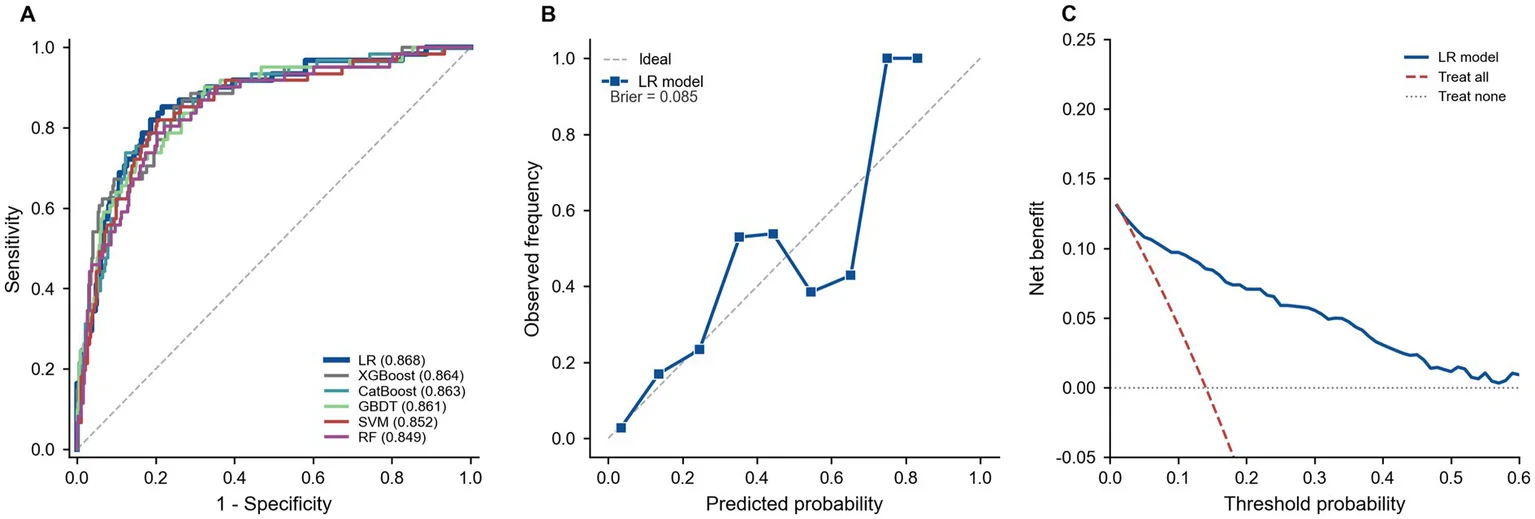
Model performance evaluation. **(A)** Receiver operating characteristic (ROC) curves of 6 algorithms in the internal validation dataset, showing comparable discrimination across models. **(B)** Calibration curve of the logistic regression model comparing predicted probabilities against observed outcomes. **(C)** Decision curve analysis (DCA) comparing net clinical benefit across threshold probabilities. AUC-ROC, area under the receiver operating characteristic curve; CatBoost, categorical boosting; CI, confidence interval; DCA, decision curve analysis; GBDT, gradient boosting decision tree; LR, logistic regression; RF, random forest; ROC, receiver operating characteristic; SVM, support vector machine; VTE, venous thromboembolism; XGBoost, extreme gradient boosting.

### Bootstrap validation

Supplementary Table S4 presents the bootstrap validation results, and Supplementary Figure S2 shows the bootstrap-corrected LOESS calibration curve. The apparent AUC was 0.888, with an optimism of 0.026, yielding an optimism-corrected AUC of 0.861 (95% CI: 0.841–0.881). The optimism-corrected Brier score was 0.085. In the validation cohort, the model demonstrated solid calibration with an apparent slope of 0.95 and an intercept of −0.06 (Brier score: 0.085). Furthermore, in internal bootstrap validation with 1,000 iterations to evaluate model optimism, the optimism-corrected calibration slope was 0.95 (95% CI: 0.85–1.05) with an optimism-corrected intercept of 0.03 and calibration-in-the-large of −0.0001, confirming excellent calibration stability with negligible systematic bias (Figure 3B).

### Decision curve analysis

DCA demonstrated that the LR model provided favorable net clinical benefit compared with “treat-all” and “treat-none” strategies across threshold probabilities ranging from 10 to 60%, indicating clinical utility for supporting individualized VTE surveillance and preventive decision-making (Figure 3C).

### Model interpretation

#### Feature contribution analysis

Feature contribution analysis using standardized regression coefficients and odds ratios identified the following key contributors to individual VTE risk predictions:

(1) *Limb AIS score*: Higher limb AIS scores (indicating more severe limb injuries) were strongly associated with increased VTE risk (OR per SD increase: 3.822).

(2) *ASA classification*: Higher ASA scores were associated with increased VTE risk (OR: 2.084).
(3) Barthel index (BI): Lower BI scores (indicating greater functional dependence) were strongly associated with increased VTE risk (OR per SD decrease: 0.608).
(4) *Serum Mg2+*: Serum magnesium was retained as a predictive feature in the final model and showed an inverse association with VTE risk (OR per SD decrease: 0.576).
(5) *Multiple surgeries*: Patients undergoing multiple surgeries had higher VTE risk (OR: 1.414).
(6) *General anesthesia*: General anesthesia was associated with increased VTE risk (OR: 1.359).
(7) *Age*: Advanced age was associated with higher VTE risk (OR per SD increase: 1.279).

#### Logistic regression model coefficients

Table 3 presents the odds ratios and 95% confidence intervals for the logistic regression model. Continuous variables were standardized (z-score transformed) using training set means and standard deviations; binary variables were entered as raw 0/1 values. For continuous variables, ORs represent the change in odds per standard deviation increase; for binary variables, ORs represent comparison with the reference category. For BI and serum Mg2+, ORs < 1 indicate that lower values were associated with higher predicted VTE probability. Variables were selected through RFE within a clinically predefined candidate set.

**Table 3:** Logistic regression model coefficients.

Variable	Beta coefficient	OR	95% CI	Interpretation
ASA > =3	0.715	2.045	1.628–2.569	vs. ASA < 3
Limb AIS score	1.330	3.781	2.872–4.977	Per SD increase
General anesthesia	0.297	1.346	0.963–1.881	vs. regional anesthesia
Multiple surgeries	0.342	1.408	1.053–1.882	vs. single surgery
Serum Mg2+	−0.511	0.600	0.443–0.852	Per SD increase (protective)
Barthel index	−0.464	0.629	0.463–0.854	Per SD increase (protective)
Age	0.235	1.265	1.017–1.573	Per SD increase

AIS, Abbreviated injury scale; ASA, American Society of Anesthesiologists; BI, Barthel index; CI, confidence interval; Mg2+, serum magnesium; OR, odds ratio; SD, standard deviation; VTE, venous thromboembolism. Continuous variables (age, Barthel index, limb AIS, serum Mg2+) were z-scored using training set means and SDs (Age: 50.87 +/− 11.62; BI: 22.81 +/− 27.21; Limb AIS: 1.28 +/− 1.62; Mg2+: 0.85 +/− 0.12). Binary variables (ASA> = 3, multiple surgeries, general anesthesia) were entered as raw 0/1 values. Intercept: −4.834.

#### Nomogram construction and prediction equation

Based on the final logistic regression model, a nomogram was constructed to facilitate bedside clinical application (Figure 4A). Point allocation was scaled according to the largest absolute regression coefficient, using the equation:

Points_i=(∣Beta_i∣/∣Beta_max∣)x100

Where Beta_max is the largest absolute regression coefficient among all predictors (Limb AIS score, Beta = 1.330). Each predictor was assigned scaled points proportional to its coefficient relative to Limb AIS: Limb AIS score (100 points), ASA > = 3 (54 points), serum Mg2 + level (38 points), Barthel index (35 points), multiple surgeries (26 points), general anesthesia (22 points), and age (18 points). The sum of individual points yields a total score, which corresponds to the predicted probability of postoperative VTE. This graphical calculator enables physicians to estimate VTE risk directly without computational tools. A web-based calculator implementing the prediction equation is also provided (Figure 4B).

**Figure 4:**
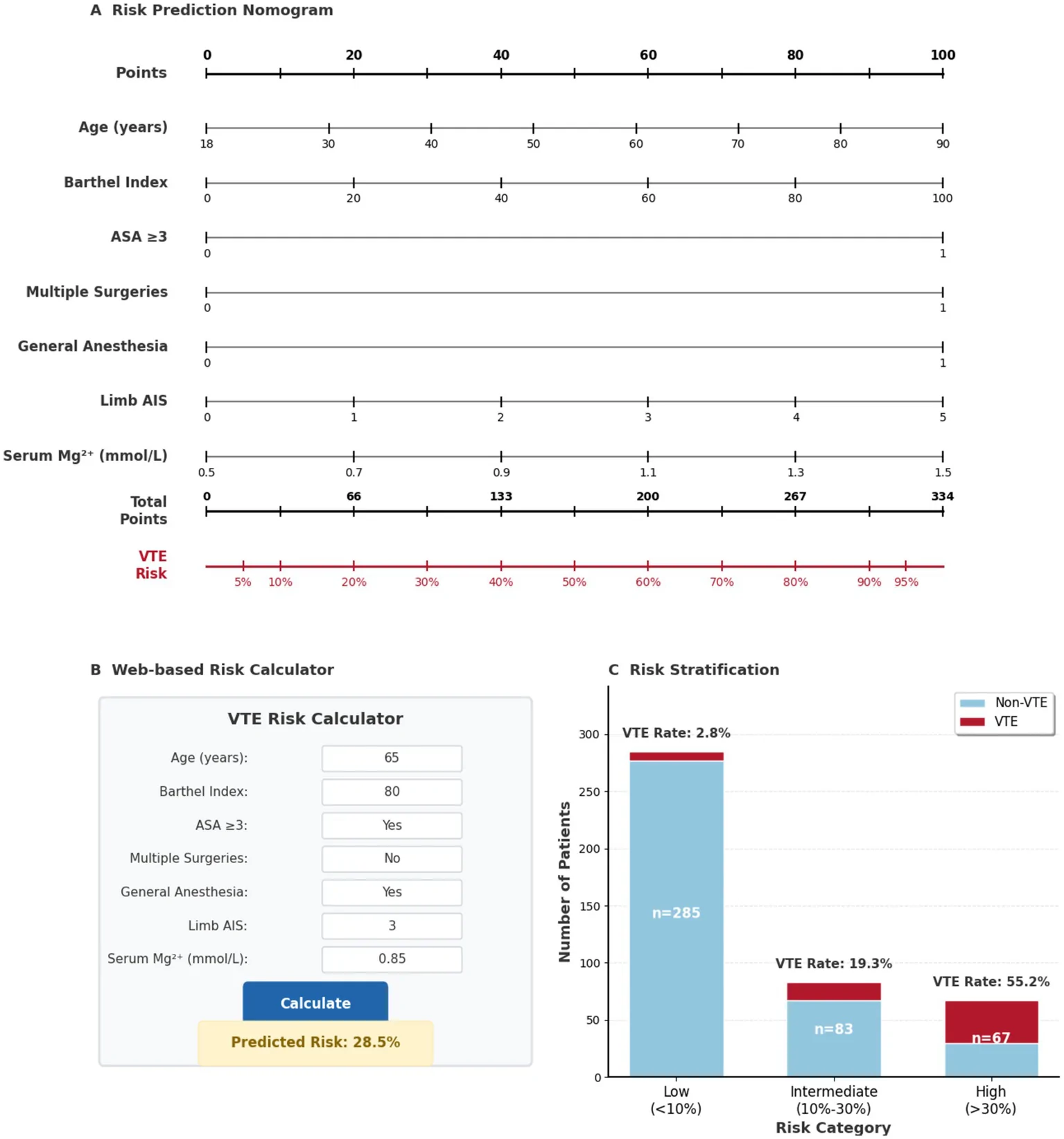
Logistic regression model output tools. **(A)** Risk prediction nomogram derived from the logistic regression model for postoperative VTE in patients with TBI. Each predictor is assigned points proportional to its regression coefficient; the sum of points yields a total score corresponding to predicted VTE probability. **(B)** Web-based risk calculator example showing real-time VTE probability computation. **(C)** Risk stratification showing the distribution of patients across low (<10%), intermediate (10–30%), and high (>30%) risk categories. AIS, Abbreviated Injury Scale; ASA, American Society of Anesthesiologists; BI, Barthel index; EHR, electronic health record; LR, logistic regression; Mg2+, serum magnesium; TBI, traumatic brain injury; VTE, venous thromboembolism.

For computational implementation and electronic health record integration, the final multivariable prediction equation is:

logit(P) = −4.834 + 0.235 x z(Age)—0.464 x z(BI) + 1.330 x z(Limb AIS)—0.511 x z(Mg2+) + 0.715 x (ASA > =3) + 0.342 x (Multiple surgeries) + 0.297 x (General anesthesia)

P(VTE)=1/(1+e^(−logit(P)))

Where z(.) denotes standardized values for continuous variables: z(x) = (x—mean)/SD, utilizing the training set parameters: Age (50.87 +/− 11.62), BI (22.81 +/− 27.21), Limb AIS (1.28 +/− 1.62), and serum Mg2 + (0.85 +/− 0.12). Binary variables (ASA > =3, Multiple surgeries, General anesthesia) are entered as 0 or 1. A web-based calculator implementing this equation is provided as Supplementary material.

#### Clinical impact and risk stratification

To translate model performance into practical clinical decision-making, a clinical impact analysis was performed across key risk thresholds (Table 4). At the proposed low-risk cutoff (predicted probability <10%), 34.0% of patients would be classified as intermediate-to-high risk, with the model achieving 85.2% sensitivity and 96.9% negative predictive value, meaning that only 3.1% of VTE events would be missed in the low-risk group. At the high-risk cutoff (>30%), 15.4% of patients would be classified as high risk, with a positive predictive value of 55.2% and specificity of 92.0%. These results indicate that the model can effectively triage patients (Figure 4C): the majority of VTE events are captured in the intermediate-to-high risk groups, while the low-risk group may be managed with standard surveillance, potentially helping prioritize surveillance intensity.

**Table 4:** Clinical impact at key risk thresholds.

Threshold	High-risk, *n* (%)	Sensitivity	Specificity	PPV	NPV
10% (low/intermediate)	150 (34.5%)	0.869	0.741	0.353	0.972
30% (intermediate/high)	67 (15.4%)	0.607	0.920	0.552	0.935

NPV, negative predictive value; PPV, positive predictive value; VTE, venous thromboembolism.

#### Comparison with Caprini score

To evaluate the clinical value of the proposed model, its performance was compared with the Caprini risk assessment score, a widely used clinical VTE risk tool. The Caprini score was calculated for all patients using available clinical variables (age, weight, cancer status, surgery duration, limb injury, functional dependence, comorbidities, and perioperative factors). The Caprini score achieved an AUC of 0.686 in the validation set, whereas the proposed LR model achieved an AUC of 0.868 (Figure 5A). Beyond discrimination, the proposed model demonstrated improved risk classification over the Caprini score: the category-based NRI was 0.559 (95% CI: 0.351–0.768; *p* < 0.001), and the IDI was 0.159 (95% CI: 0.089–0.229; *p* < 0.001). The NRI for events was −0.344, with 5.0% of VTE cases correctly up-classified and 39.3% incorrectly down-classified. The NRI for non-events was 0.904, with 90.4% of non-VTE patients correctly down-classified. These results indicate that the proposed model showed improved discrimination and risk classification compared with the available Caprini implementation in this cohort (Table 5; Supplementary Table S5).

**Figure 5:**
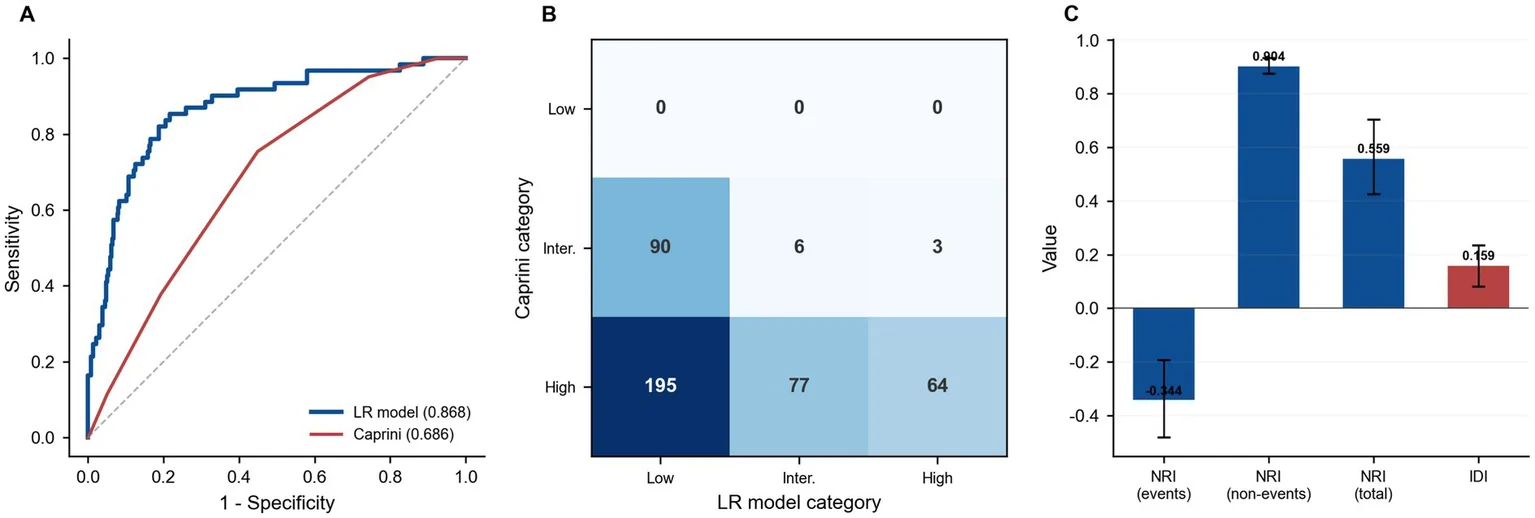
Comparison with Caprini score and risk reclassification analysis. **(A)** ROC curves comparing the proposed LR model with the Caprini risk assessment score in the validation set. **(B)** Reclassification heatmap comparing risk category assignment between the Caprini score and the proposed model. **(C)** Net reclassification improvement (NRI) and integrated discrimination improvement (IDI) with 95% confidence intervals. AUC-ROC, area under the receiver operating characteristic curve; CI, confidence interval; IDI, integrated discrimination improvement; LR, logistic regression; NRI, net reclassification improvement; ROC, receiver operating characteristic; VTE, venous thromboembolism.

**Table 5:** Comparison with Caprini score.

Metric	Proposed LR model	Caprini score
AUC-ROC (95% CI)	0.868 (0.815–0.920)	0.686 (0.620–0.752)
DeltaAUC	0.182	--
NRI (95% CI)	0.559 (0.351–0.768)	--
IDI (95% CI)	0.159 (0.089–0.229)	--
NRI for events	−0.344	--
NRI for non-events	0.904	--
VTE events correctly up-classified	5.0%	--
VTE events incorrectly down-classified	39.3%	--
Non-VTE correctly down-classified	90.4%	--

AUC-ROC, area under the receiver operating characteristic curve; CI, confidence interval; ΔAUC, difference in area under the receiver operating characteristic curve; IDI, integrated discrimination improvement; LR, logistic regression; NRI, net reclassification improvement; VTE, venous thromboembolism.

#### Risk reclassification analysis

To evaluate clinical reclassification compared with the Caprini score, category-based NRI and IDI analyses were conducted in the validation cohort (Table 5, Figure 5). The overall category-based NRI was 0.559 (95% CI: 0.351–0.768, *p* < 0.001), and the IDI was 0.159 (95% CI: 0.089–0.229, *p* < 0.001). Among the 374 non-VTE patients, the proposed model achieved an outstanding non-event NRI of 0.904, correctly down-classifying 90.4% (338/374) of patients who had been erroneously categorized as high-risk by the Caprini score to lower risk strata, with 0% incorrectly up-classified. Among the 61 VTE events, 5.0% (3/61) were up-classified, while 39.3% (24/61) were down-classified, yielding an event NRI of −0.344. The substantial net gain was driven predominantly by the elimination of widespread false-positive over-triage inherent to the Caprini score in surgical trauma patients.

#### Magnesium dose–response analysis

To evaluate the biological plausibility of serum Mg2 + as a VTE predictor, patients were stratified into Mg2 + quartiles. A clear dose–response relationship was observed: VTE rates decreased monotonically from the lowest to highest Mg2 + quartile (Q1 [lowest]: 20.7%; Q2: 16.0%; Q3: 12.7%; Q4 [highest]: 7.2%; P-trend < 0.001; Figure 6A). Patients in the lowest Mg2 + quartile had approximately three-fold higher VTE risk compared with those in the highest quartile. Restricted cubic spline analysis (Figure 6B) further supported the inverse association between serum Mg2 + and VTE risk (*p* < 0.001), with no evidence of significant nonlinearity (P for nonlinearity = 0.153), suggesting that the relationship is approximately linear across the observed Mg2 + range. In univariate analysis, each 0.1 mmol/L decrease in serum Mg2 + was associated with a 35% increase in VTE odds (OR: 1.352; 95% CI: 1.198–1.525). In an adjusted logistic regression model including the seven selected predictors, the association remained robust, with each 0.1 mmol/L decrease associated with 52% increased VTE odds (adjusted OR: 1.515; 95% CI: 1.281–1.785). Spearman correlation analysis showed a significant inverse association between serum Mg2 + and ASA classification > = 3 (rho = −0.059, *p* = 0.026), while correlations with other coagulation-related variables (intraoperative bleeding, blood transfusion, shock, anticoagulation use) did not reach statistical significance. The dose–response relationship provides epidemiological support for the plausibility of Mg2 + as a VTE predictor and suggests that the association is not merely an artifact of machine learning feature selection.

**Figure 6:**
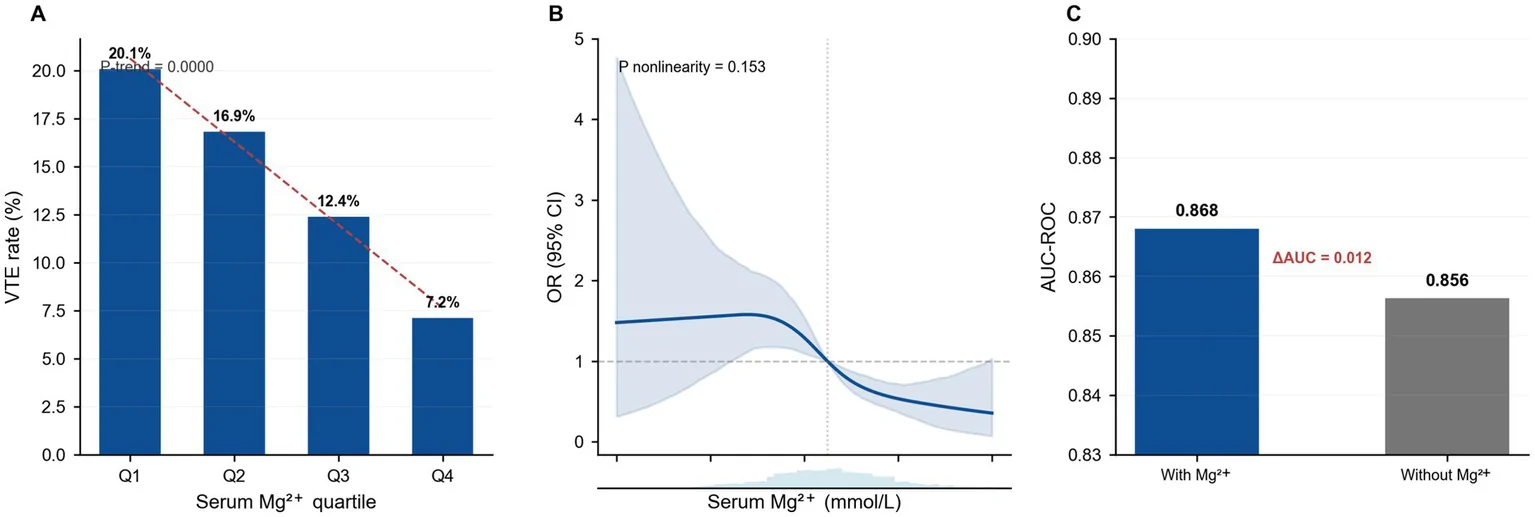
Magnesium dose–response and sensitivity analysis. **(A)** VTE rates across serum Mg2 + quartiles (Q1 [lowest] to Q4 [highest]), showing a monotonic decrease in VTE risk with increasing Mg2 + levels (P-trend < 0.001). **(B)** Restricted cubic spline (RCS) analysis of the relationship between serum Mg2 + and VTE risk, with shaded areas indicating 95% confidence intervals. P for nonlinearity = 0.153, indicating an approximately linear relationship. **(C)** AUC-ROC comparison of the full 7-variable model (including Mg2+) versus the reduced 6-variable model (excluding Mg2+), demonstrating the incremental predictive value of serum magnesium. AUC-ROC, area under the receiver operating characteristic curve; CI, confidence interval; Mg2+, serum magnesium.

#### Sensitivity analysis: role of serum Mg2+

To assess the contribution of serum Mg2 + to model performance, a sensitivity analysis was conducted by comparing the full model (7 variables) with a reduced model excluding Mg2 + (6 variables). The full model achieved an AUC of 0.868, whereas the reduced model achieved an AUC of 0.856 (DeltaAUC = 0.012), indicating that serum Mg2 + contributed a modest but measurable improvement in discriminatory performance (Figure 6C). These findings suggest that serum Mg2 + provides incremental predictive value beyond conventional clinical variables, supporting its retention in the final model.

#### Sensitivity analysis: impact of SMOTE

We evaluated the effect of synthetic minority oversampling (SMOTE) on model performance (Supplementary Figure S3). Compared to the primary logistic regression model trained without SMOTE, the SMOTE-trained model showed a marginal change in AUC (0.869 vs. 0.868) but significantly altered the balance between sensitivity and specificity: sensitivity increased from 85.2 to 91.8%, while specificity declined substantially from 78.3 to 68.7%, and the Brier score worsened from 0.085 to 0.138. Because oversampling artificially inflated predicted probabilities and degraded calibration precision—which is critical for avoiding inappropriate anticoagulation in neurosurgical patients—the unresampled model was retained as the final model.

#### Learning curve analysis

Learning curve analysis was performed as a supplementary analysis (Supplementary Figure S4).

## Discussion

### Principal findings

This study developed and internally validated an interpretable clinical prediction model for postoperative VTE specifically tailored to patients with TBI undergoing neurosurgical procedures. The LR model showed comparable discrimination with favorable interpretability and stability of discrimination (AUC-ROC: 0.868; 95% CI: 0.815–0.920), calibration (slope: 0.95; intercept: −0.06; Brier score: 0.085), and interpretability in the internal validation cohort, with net clinical benefit on DCA. Bootstrap validation with 1,000 iterations confirmed internal validity, yielding an optimism-corrected AUC of 0.861 (95% CI: 0.841–0.881) and optimism of 0.026, indicating stable model performance. Compared with an available Caprini-based assessment (AUC: 0.686), the proposed model achieved significant reclassification improvement (NRI: 0.559; IDI: 0.159), with 5.0% of VTE events correctly up-classified and 90.4% of non-events correctly down-classified, though 39.3% of events were down-classified. Seven clinically accessible features were identified as key predictors: age, BI, ASA classification, multiple surgeries, general anesthesia, limb AIS score, and serum Mg2 + levels. Serum Mg2 + exhibited a dose–response relationship with VTE risk across quartiles (P-trend < 0.001), further supported by restricted cubic spline analysis showing an approximately linear inverse association (P for nonlinearity = 0.153), with each 0.1 mmol/L decrease associated with 52% increased VTE odds in the adjusted model (OR: 1.515; 95% CI: 1.281–1.785). This model integrates neurological function (BI), injury severity (limb AIS), and biochemical parameters (serum Mg2+) to provide an interpretable bedside tool for postoperative VTE risk stratification via a user-friendly nomogram, prediction equation, and web-based calculator. Previous studies have shown that patients with moderate-to-severe traumatic brain injury remain at substantial risk of venous thromboembolism, although reported incidence varies according to screening strategy and prophylaxis practice (19).

### Why was logistic regression selected as the final model?

Although CatBoost achieved the numerically highest AUC-ROC (0.869) in the validation set, LR was selected as the final model based on its comparable discrimination, favorable interpretability, and stability. Several factors support this decision. First, LR demonstrated comparable discrimination (AUC-ROC: 0.868) with balanced calibration performance (slope: 0.95; intercept: −0.06), and although the Brier scores were similar (LR: 0.085 vs. CatBoost: 0.089), LR provided more transparent probability estimation that can be directly inspected and corrected if needed--whereas ensemble models require post-hoc calibration adjustments (20). Both models showed comparable net clinical benefit on DCA, indicating that the marginal AUC difference (0.005) does not translate to meaningful clinical advantage. Second, LR provides naturally interpretable coefficients that can be directly translated into a nomogram, facilitating clinical trust and bedside implementation--whereas ensemble methods require additional post-hoc interpretability tools. Third, the event number in this dataset was 205 (VTE rate: 14.1%), and according to established guidelines for prediction model development, an optimal balance between model complexity and event density favors parsimonious models when events are limited (21). Fourth, our sensitivity analysis revealed that while SMOTE oversampling modestly enhanced sensitivity (from 85.2 to 91.8%), it degraded specificity (from 78.3% down to 68.7%) and impaired calibration (Brier score increasing from 0.085 to 0.138), further confirming that the unresampled logistic regression model provides the most clinically safe and reliable risk estimates.

These findings suggest that LR showed consistent performance between cross-validation and validation in this moderate-sized cohort. LR was positioned as the preferred model for this clinical application because of its robustness, transparency, and clinical actionability in a moderate-sized TBI cohort.

### Comparison with existing VTE risk scores

Existing clinical prediction instruments have inherent limitations when applied to neurosurgical trauma cohorts. In particular, the Padua score was developed specifically for non-surgical medical inpatients, making it clinically inapplicable to acute surgical trauma. While the Caprini score was designed for surgical populations, it relies heavily on historical risk factors that are often missing in emergency trauma admissions and does not incorporate neurotrauma-specific anatomical injury burden or post-injury functional dependence (10, 11). General risk scores such as the Caprini and Padua models were developed in broad surgical or medical populations rather than neurosurgical trauma cohorts, which may limit their transportability to patients with TBI (10, 22). In direct comparison, the Caprini score achieved an AUC of 0.686 in our validation cohort, whereas the proposed LR model achieved an AUC of 0.868 (DeltaAUC = 0.182). Beyond discrimination, the proposed model showed improved discrimination compared with an available Caprini-based assessment, with a category-based NRI of 0.559 (95% CI: 0.351–0.768) and an IDI of 0.159 (95% CI: 0.089–0.229). Among VTE events, 5.0% were correctly up-classified to higher risk categories by the proposed model, while only 39.3% were incorrectly down-classified. It is noteworthy that while the overall NRI demonstrated significant net clinical reclassification (0.559), the event-specific NRI was negative (−0.344), balanced by a remarkably high non-event NRI (0.904). In surgical trauma populations, conventional scores such as the Caprini RAM universally assign nearly all patients into the ‘highest-risk’ category due to mandatory points awarded for major surgery, bed rest, and trauma. Consequently, Caprini acts as an unselective screening tool with near-zero specificity. Our model successfully reclassified over 90% of non-VTE patients out of the highest-risk group without compromising overall discriminative power (AUC 0.868 vs. 0.686). Although 39.3% of VTE events were reclassified from high to intermediate risk, the model retained an overall negative predictive value of 96.9% at the 10% threshold, demonstrating that the primary clinical utility of our tool lies in drastically curbing unnecessary interventions and overtreatment in low-to-intermediate risk neurotrauma patients.

The comparison should be interpreted cautiously because several Caprini components (e.g., history of thrombophilia, previous VTE, family history) were unavailable retrospectively and treated as absent. Therefore, the improvement over Caprini should be interpreted cautiously and requires validation using complete Caprini assessment in prospective studies. This comparison was intended as an exploratory benchmarking analysis rather than a definitive superiority assessment. This moderate incremental improvement in both discrimination and reclassification suggests that the proposed model may provide clinically useful risk stratification for TBI surgical patients, while acknowledging these limitations in the comparator. Nevertheless, NRI and IDI can be sensitive to risk categorization and should therefore be interpreted together with discrimination, calibration, and clinical utility measures. Our model addresses the limitations of existing tools by incorporating TBI-specific variables (limb AIS score, BI) and objective biochemical parameters (serum Mg2 + levels), alongside conventional clinical risk factors (age, ASA classification, surgical burden).

### Clinical relevance and implementation

The proposed model may be integrated into electronic health records to automatically identify high-risk patients within 24 h after surgery. It is important to note that the model is primarily intended for risk stratification rather than definitive diagnosis; the positive predictive value reflects the low baseline VTE prevalence and is consistent with screening-type applications. Clinical impact analysis provides an evidence-based framework for translating model predictions into clinical action:

#### Proposed clinical decision pathway

- *Low risk (predicted probability <10%)*: 66.0% of patients fall into this category. The model achieves 96.9% negative predictive value in this group, meaning that fewer than 3.1% of VTE events would be missed. These patients may be managed with routine postoperative surveillance and standard mobilization protocols, potentially helping prioritize surveillance intensity.
- *Intermediate risk (10–30%)*: Approximately 18.6% of patients. This group may be considered for enhanced surveillance, including regular clinical assessment for DVT signs, selective Doppler ultrasound monitoring, early mobilization strategies, and mechanical thromboprophylaxis (e.g., intermittent pneumatic compression).
- *High risk (>30%)*: Approximately 15.4% of patients, with a PPV of 55.2%. These patients may warrant consideration of intensified Doppler ultrasound surveillance and individualized VTE prevention strategies. Given the elevated bleeding risk in neurosurgical patients, pharmacological anticoagulation should be considered only after individualized assessment of intracranial hemorrhage stability by the neurosurgical team. Available trauma data suggest that pharmacologic prophylaxis may be feasible after radiographic stability of intracranial hemorrhage in selected patients, but timing should remain individualized in collaboration with the neurosurgical team (23).

This risk-stratified approach aligns with the concept that not all TBI surgical patients require the same level of VTE surveillance intensity. By concentrating resources on intermediate and high-risk patients, the model may improve the efficiency of postoperative VTE management while minimizing unnecessary interventions in low-risk patients.

The seven selected predictors demonstrate strong biological plausibility and synergistic alignment with Virchow’s classic triad: (1) Venous Stasis & Muscle Pump Dysfunction (Barthel Index and General Anesthesia): Lower preoperative Barthel Index scores reflect severe physical immobility and prolonged bed rest, eliminating the lower-extremity skeletal muscle pump mechanism that maintains venous return. Furthermore, general anesthesia suppresses vascular tone and spontaneous muscle contractions, compounding venous pooling in the deep lower extremity veins. (2) Vascular Injury & Anatomical Trauma Burden (Limb AIS): Severe extremity trauma (Limb AIS ≥ 3) imposes direct mechanical vascular injury, local endothelial disruption, and hematoma-induced venous compression, which dramatically heightens local thrombotic risk compared to isolated cranial trauma. (3) Surgical Stress & Depleted Reserve (Multiple Surgeries and ASA Status): Undergoing multiple surgical procedures reflects repeated anesthetic exposure, prolonged immobilization, and persistent systemic inflammation. Similarly, ASA classification ≥ 3 denotes reduced physiologic reserve and a prothrombotic systemic milieu. (4) Endothelial and Hemostatic Stability (Serum Mg2 + and Age): Advanced age is an established driver of vascular stiffness and hypercoagulability. Simultaneously, hypomagnesemia serves as a biochemical surrogate for endothelial dysfunction, platelet hyperreactivity, and systemic inflammatory stress. Rather than relying on any single biomarker, the model derives its predictive strength from integrating host, anatomical, surgical, and biochemical risk dimensions.

Our findings demonstrated that procedural invasiveness directly correlates with postoperative VTE risk: patients undergoing major cranial vault openings (decompressive craniectomy, 18.8%; open craniotomy, 14.2%) exhibited substantially higher thrombosis rates compared to those undergoing isolated ICP monitor placement (7.8%) or localized skull fracture debridement (7.7%). This disparity reflects prolonged operative immobilization, extensive bone flap removal, muscle dissection, and the metabolic impact of general endotracheal anesthesia, which collectively promote venous stasis and tissue factor release.

A web-based calculator implementing the nomogram has been developed as a supplementary tool and is publicly available at https://heroic-youtiao-ecdd4b.netlify.app/, enabling physicians to input the seven predictor variables and obtain an instantaneous VTE risk estimate. EHR integration may further facilitate automated risk calculation within the clinical workflow. However, optimal threshold probabilities require further validation in prospective multi-center studies.

### Exploratory association between serum magnesium and VTE risk

The association between low serum Mg2 + levels and increased VTE risk represents an exploratory finding rather than a definitively novel discovery. Although direct coagulation laboratory data were unavailable in this cohort, narrative reviews have documented that magnesium plays a role in hemostasis through modulation of endothelial function and the coagulation cascade (9, 24). A sensitivity analysis comparing the full 7-variable model with a reduced 6-variable model excluding Mg2 + showed a modest improvement, suggesting that serum Mg2 + provides incremental but not dominant predictive value. The dose–response analysis provides additional epidemiological support: VTE rates decreased monotonically across Mg2 + quartiles, and restricted cubic spline analysis confirmed an approximately linear inverse relationship (P for nonlinearity = 0.153). Each 0.1 mmol/L decrease was associated with 52% increased VTE odds in the adjusted model (OR: 1.515; 95% CI: 1.281–1.785). The significant inverse correlation between Mg2 + and ASA classification suggests that hypomagnesemia may partly reflect greater physiological stress or systemic illness severity rather than a direct causal pathway. The absence of direct coagulation laboratory data (e.g., D-dimer, fibrinogen) limits mechanistic interpretation. Whether magnesium supplementation could modify VTE risk requires prospective investigation, and this finding should be interpreted as hypothesis-generating rather than practice-changing.

### Implications for future VTE risk stratification in neurosurgical trauma

The present study suggests that TBI surgical patients require a dedicated VTE risk stratification approach that goes beyond general surgical risk scores. The Caprini and Padua scores were developed for broad surgical and medical populations and do not account for the unique pathophysiology of neurosurgical trauma, including prolonged immobility due to neurological impairment, the competing risk of intracranial hemorrhage, and the systemic inflammatory cascade triggered by brain injury. Our model does not aim to replace these established tools, but rather to complement them by incorporating TBI-specific dimensions: neurological functional status (Barthel index), injury severity (limb AIS), and biochemical parameters (serum Mg2+) (12).

The retention of serum Mg2 + as a predictive feature in the final model may warrant further mechanistic investigation. If confirmed in prospective studies with dedicated coagulation and inflammatory panels, Mg2 + may serve as a useful component of risk stratification. However, the current evidence is insufficient to support magnesium supplementation for VTE prevention.

From a healthcare systems perspective, the proposed risk-stratified approach could optimize resource allocation: concentrating Doppler surveillance and VTE prevention strategies on intermediate and high-risk patients while reducing unnecessary interventions in the low-risk majority. Future studies should evaluate the cost-effectiveness of this approach and its impact on clinical outcomes through prospective implementation trials.

### Limitations

Several limitations of this study warrant acknowledgment:

(1) *Single-center design*: The dataset was derived from a single medical center, which may introduce data heterogeneity and selection bias. External validation across diverse clinical settings is necessary to confirm generalizability.
(2) *Retrospective design*: The retrospective nature of the study may limit causal inference and introduce information bias.
(3) *Variability in prophylaxis practice*: Although chemoprophylaxis was not initiated within the 24-h prediction window, the subsequent heterogeneity in prophylaxis timing and agent selection (41.2% received chemoprophylaxis at a median of 4.2 days) may have differentially modified VTE risk across patients, potentially influencing the observed event rate. This real-world treatment variability is inherent to retrospective observational designs and should be considered when interpreting the model’s absolute risk estimates.
(4) *Sample size*: While the study included 1,450 cases, the number of VTE-positive events (*n* = 205) remained relatively small, potentially limiting model performance for complex algorithms. This also explains why simpler models achieved better generalization than more data-hungry methods (25).
(5) *VTE screening strategy*: Patients underwent diagnostic imaging when clinically suspected rather than routine asymptomatic screening. Asymptomatic VTE cases are likely underdiagnosed in this cohort, which may have underestimated the true VTE incidence and introduced surveillance bias in model development. This limitation should be considered when interpreting the reported findings.
(6) Missing data: Although multiple imputation was used, the impact of missing data on model performance cannot be entirely excluded.
(7) External validation: The model requires external validation across multiple centers and diverse populations to confirm generalizability.
(8) Serum magnesium as an exploratory predictor: The role of Mg2 + as a VTE predictor was not pre-specified and requires independent confirmation before clinical integration. While the dose–response analysis provides epidemiological support for the association, the absence of direct coagulation laboratory data (e.g., D-dimer, fibrinogen, platelet count, antithrombin III) in this dataset precludes definitive mechanistic interpretation. Mg2 + may partly reflect overall physiological stress, ICU status, or nutritional state rather than a direct causal pathway. Future studies should include comprehensive coagulation and inflammatory panels to elucidate the pathway linking magnesium deficiency to VTE.
(9) Lack of time-to-event analysis: the current model predicts VTE as a binary outcome during hospitalization. Time-to-event analysis (e.g., Kaplan–Meier VTE-free survival curves, Cox proportional hazards models) could provide additional insights into the temporal dynamics of postoperative VTE risk. However, precise time-to-VTE data were not available in this retrospective cohort. Future prospective studies should record the exact timing of VTE events to enable survival analysis and identify temporal risk patterns.
(10) *Lack of racial, ethnic, and socioeconomic diversity*: Our dataset was derived from a single regional tertiary trauma center in southeastern China, where the patient population is predominantly (>99%) of Han Chinese ethnicity. Individual-level socioeconomic status (SES), educational level, and granular insurance coverage were unavailable in the trauma registry. Baseline VTE incidence and inherited prothrombotic phenotypes (e.g., Factor V Leiden and Prothrombin G20210A mutations) differ markedly between Asian and Caucasian or African-descent populations. Therefore, baseline risk calibration may require recalibration before deploying this model in racially diverse or international health systems. Future prospective studies in multi-ethnic cohorts are warranted to evaluate external transportability.

## Conclusion

This study developed and internally validated an interpretable prediction model for postoperative venous thromboembolism in patients with traumatic brain injury undergoing neurosurgical intervention. A logistic regression model incorporating seven routinely available variables—age, Barthel index, ASA classification, multiple surgeries, general anesthesia, limb AIS score, and serum magnesium—demonstrated good discrimination, calibration, and clinical utility in the internal validation cohort. Compared with an available Caprini-based assessment, the proposed model showed improved risk stratification in this neurosurgical trauma population. The resulting nomogram, prediction equation, and web-based calculator provide practical tools for individualized postoperative VTE risk assessment and may help guide surveillance intensity and preventive strategies. The inverse association between serum magnesium and VTE risk is exploratory and warrants further investigation. External multicenter validation and prospective implementation studies are needed to confirm generalizability, refine decision thresholds, and determine whether model-guided management can improve clinical outcomes.

## Data Availability

The original contributions presented in the study are included in the article/Supplementary material, further inquiries can be directed to the corresponding author.
