# Supplementary material for: Development and internal validation of a clinical prediction model for postoperative venous thromboembolism after neurosurgical intervention in patients with traumatic brain injury
Source: Front Neurol. 2026 Oct 12;17:1953953. doi: 10.3389/fneur.2026.1953953 (PMC13701100; doi:10.3389/fneur.2026.1953953)
Supplement: Supplementary file 1 [file Supplementary_file_1.docx]

**Supplementary Figure Legends**

**Supplementary Figure S1.** Missing data pattern visualization. The heatmap shows the pattern of missing data across variables in the study dataset, with darker colors indicating higher proportions of missing values. Variables are ordered by the proportion of missing data.

**Supplementary Figure S2.** Bootstrap-corrected LOESS calibration curve. The blue solid line represents the apparent calibration (model evaluated on the training data), and the red dashed line represents the optimism-corrected calibration (adjusted via 1000 bootstrap resamples). The black diagonal line represents ideal calibration. The histogram at the bottom shows the distribution of predicted probabilities. LOESS: locally estimated scatterplot smoothing.

**Supplementary Figure S3.** SMOTE sensitivity analysis. Comparison of model performance metrics (AUC-ROC, sensitivity, specificity, Brier score) between the primary model (without SMOTE) and the SMOTE-trained model, demonstrating the trade-off between discrimination improvement and calibration deterioration.

**Supplementary Figure S4.** Learning curve analysis comparing logistic regression (LR), random forest (RF), and gradient boosting decision tree (GBDT) across training set sizes (10%-90% of full dataset, 20 repetitions per size). Shaded areas represent standard deviation. AUC: area under the receiver operating characteristic curve.
