# Supplementary material for: Development and internal validation of a clinical prediction model for postoperative venous thromboembolism after neurosurgical intervention in patients with traumatic brain injury
Source: Front Neurol. 2026 Oct 12;17:1953953. doi: 10.3389/fneur.2026.1953953 (PMC13701100; doi:10.3389/fneur.2026.1953953)
Supplement: Supplementary file 2 [file Table_1.docx]

# Supplementary Methods

## Detailed Algorithm Configurations and Hyperparameter Optimization

### Machine Learning Algorithms

Six machine learning algorithms were evaluated in this study:

1. **Logistic Regression (LR)**: Linear model with L2 (Ridge) regularization. Regularization strength parameter C was optimized over [0.001, 0.01, 0.1, 1.0, 10.0, 100.0]. Solver: L-BFGS (Limited-memory Broyden-Fletcher-Goldfarb-Shanno). Maximum iterations: 1000. Convergence tolerance: 1e-4.
2. **Random Forest (RF)**: Bagging-based ensemble method. Number of trees optimized through grid search (maximum 500 trees). Splitting criterion: Gini impurity.
3. **Gradient Boosting Decision Tree (GBDT)**: Sequential boosting method. Learning rate and number of estimators optimized. Maximum iterations: 1000 with early stopping (patience=50).
4. **Extreme Gradient Boosting (XGBoost)**: Optimized gradient boosting implementation. Learning rate and tree depth optimized. Maximum iterations: 1000 with early stopping.
5. **Support Vector Machine (SVM)**: Kernel-based non-parametric method with RBF kernel. Tolerance: 1e-4. Maximum iterations: 10000. Probability estimates via Platt scaling.
6. **Categorical Boosting (CatBoost)**: Categorical-optimized boosting method. Learning rate and tree depth optimized. Maximum iterations: 1000 with early stopping.

### Hyperparameter Optimization

All hyperparameters were optimized using five-fold stratified cross-validation with exhaustive grid search on the training set only. The grid search space was designed to cover a range of model complexities while maintaining computational feasibility. The best hyperparameters were selected based on the highest mean cross-validation AUC-ROC across the five folds.

**Table. Hyperparameter Grid Search Space**

| **Algorithm** | **Hyperparameters** | **Grid Values** |
| --- | --- | --- |
| LR | C (regularization strength) | [0.001, 0.01, 0.1, 1.0, 10.0, 100.0] |
| RF | n_estimators, max_depth | [100, 200, 300, 400, 500]; [None, 5, 10, 15] |
| GBDT | n_estimators, learning_rate, max_depth | [100, 200, 300]; [0.01, 0.05, 0.1]; [3, 5, 7] |
| XGBoost | n_estimators, learning_rate, max_depth | [100, 200, 300]; [0.01, 0.05, 0.1]; [3, 5, 7] |
| SVM | C, gamma | [0.1, 1.0, 10.0]; ['scale', 'auto'] |
| CatBoost | iterations, learning_rate, depth | [100, 200, 300]; [0.01, 0.05, 0.1]; [4, 6, 8] |

## Data Preprocessing Details

### Missing Data Handling

Missing data were handled using multiple imputation by chained equations (MICE) with predictive mean matching for continuous variables and logistic regression imputation for binary variables. The number of imputations was set to 5, and results were pooled using Rubin's rules.

**Missing Data Assessment:**

- Variables with >20% missing data were excluded from analysis
- Missing completely at random (MCAR) assumption tested using Little's test
- If MCAR rejected (P<0.05), missing at random (MAR) assumed
- Imputation model included all candidate predictors and outcome variable

### Feature Standardization

All continuous predictors were standardized using z-score transformation:

- z(x) = (x - mean) / SD
- Standardization parameters computed from training set only
- Training set statistics:

- Age: 50.87 ± 11.62 years

- Barthel index: 22.81 ± 27.21

- Limb AIS: 1.28 ± 1.62

- Serum Mg2+: 0.85 ± 0.12 mmol/L

### Information Leakage Prevention

All preprocessing steps were performed within a scikit-learn Pipeline framework:

7. Imputation model fitted only on training data within each CV fold
8. Fitted parameters applied to transform validation data
9. Standardization parameters computed from training fold only
10. RFE feature selection performed within each CV fold

## Detailed Model Evaluation Metrics

### Discrimination Metrics

- **AUC-ROC**: Area under receiver operating characteristic curve (primary metric)
- **Accuracy**: (TP+TN)/(TP+TN+FP+FN)
- **Sensitivity**: TP/(TP+FN)
- **Specificity**: TN/(TN+FP)
- **PPV**: TP/(TP+FP)
- **NPV**: TN/(TN+FN)
- **F1-score**: 2×PPV×Sensitivity/(PPV+Sensitivity)
- **Optimal threshold**: Youden index (J = Sensitivity + Specificity - 1)

### Calibration Metrics

- **Brier score**: Mean squared difference between predicted and observed outcomes
- **Calibration slope**: Slope of logistic regression of observed on predicted logits (ideal=1)
- **Calibration intercept**: Intercept of logistic regression (ideal=0)
- **Calibration-in-the-large**: Mean predicted probability vs observed event rate

### Clinical Utility

- **Decision Curve Analysis (DCA)**: Net benefit across threshold probabilities (0-100%)
- **Clinical Impact Analysis**: High-risk patients identified, true/false positives at key thresholds

## Bootstrap Validation Procedure

Internal validity was assessed using bootstrap resampling with 1000 iterations:

11. For each iteration (i = 1 to 1000):

- Draw bootstrap sample of same size as training set (n=1015) with replacement

- Refit entire modeling pipeline (imputation, standardization, RFE, model training)

- Evaluate on bootstrap sample → apparent performance (optimistic)

- Evaluate on original training set → test performance

- Calculate optimism = apparent - test performance

12. Optimism-corrected performance = apparent performance - mean optimism across 1000 iterations
13. Bootstrap-corrected LOESS calibration curve generated by averaging predicted probabilities across all iterations

## Sensitivity Analyses

### 1. Imputation Method Comparison

- Multiple imputation by chained equations (MICE) - primary method
- Mean imputation
- Median imputation
- K-nearest neighbor imputation

### 2. Train-Validation Split Ratios

- 70:30 split (primary)
- 60:40 split
- 80:20 split

### 3. SMOTE Oversampling Analysis

- SMOTE (primary sensitivity analysis)
- Borderline-SMOTE
- ADASYN (Adaptive Synthetic Sampling)

### 4. Regularization Strength

- C = 0.1 (strong regularization)
- C = 1.0 (primary)
- C = 10.0 (weak regularization)

## Reproducibility

- Fixed random seeds: random_state=42 (scikit-learn), seed=42 (XGBoost, CatBoost)
- Single Python script implementing entire pipeline
- scikit-learn Pipeline objects ensure consistent preprocessing
- Source code available from corresponding author

## Computational Environment

- Hardware: Intel Xeon Gold 6248R CPU (3.00 GHz, 48 cores), 192 GB RAM
- Operating System: Ubuntu 20.04 LTS
- Total computational time: ~45 minutes for entire modeling pipeline
